# Reduced Polymorphism Associated with X Chromosome Meiotic Drive in the Stalk-Eyed Fly *Teleopsis dalmanni*

**DOI:** 10.1371/journal.pone.0027254

**Published:** 2011-11-08

**Authors:** Sarah J. Christianson, Cara L. Brand, Gerald S. Wilkinson

**Affiliations:** Department of Biology, University of Maryland, College Park, Maryland, United States of America; American Museum of Natural History, United States of America

## Abstract

Sex chromosome meiotic drive has been suggested as a cause of several evolutionary genetic phenomena, including genomic conflicts that give rise to reproductive isolation between new species. In this paper we present a population genetic analysis of X chromosome drive in the stalk-eyed fly, *Teleopsis dalmanni*, to determine how this natural polymorphism influences genetic diversity. We analyzed patterns of DNA sequence variation at two X-linked regions (comprising 1325 bp) approximately 50 cM apart and one autosomal region (comprising 921 bp) for 50 males, half of which were collected in the field from one of two allopatric locations and the other half were derived from lab-reared individuals with known brood sex ratios. These two populations are recently diverged but exhibit partial postzygotic reproductive isolation, i.e. crosses produce sterile hybrid males and fertile females. We find no nucleotide or microsatellite variation on the drive X chromosome, whereas the same individuals show levels of variation at autosomal regions that are similar to field-collected flies. Furthermore, one field-caught individual collected 10 years previously had a nearly identical X haplotype to the drive X, and is over 2% divergent from other haplotypes sampled from the field. These results are consistent with a selective sweep that has removed genetic variation from much of the drive X chromosome. We discuss how this finding may relate to the rapid evolution of postzygotic reproductive isolation that has been documented for these flies.

## Introduction

Sex chromosome meiotic drive is a selfish genetic system characterized by non-Mendelian transmission of sex chromosomes [1]. In the most common type, biased transmission of the X occurs at the expense of the Y [2], [3]–[7]. In these systems, the driving X chromosome (*X^D^*) outcompetes non-driving X chromosomes (*X^ST^*) and should proceed to fixation if unimpeded by selection or unmodified by other loci as the population sex ratio becomes increasingly biased towards females [8]. However, multiple levels of selection can act on drive and prevent fixation. For example, in a female-biased population females that mate with non-driving males have higher fitness because they produce more sons [9]. Traits indicative of a male's drive status may then evolve, allowing females to choose mates that produce a favorable sex ratio [10], [11]. Additionally, selection to balance the sex ratio favors the spread of autosomal and Y-linked drive suppressors [6], [12]–[16]. A driving X chromosome also faces selection to increase its own success and may accumulate modifiers that enhance drive [17]. In general, evolution of drive, associated modifiers and suppressors can lead to complete masking of the drive phenotype [18]–[20], cycles, or a polymorphism [21]–[23].

A drive polymorphism can be stabilized if females that carry drive have reduced fitness or are overdominant [24], [25] or if frequency dependent selection operates on drive males strongly enough to offset segregation distortion [26]. While it is uncommon to find a population with a significant proportion of males harboring unsuppressed drive [4], [27], studies using lab and field-reared flies indicate that viability and fertility selection operate against SR in both sexes in some species of flies [24], [25], [28], [29]. Reduced fertility of drive males is expected given that their Y-bearing sperm fail to develop and has been frequently observed [27], [29].

Selection on drive can influence DNA polymorphism in several ways. Positive selection on either drive, modifier or suppressor loci is expected to lead to genetic hitchhiking at linked loci and reduce polymorphism [30]–[32]. For example, recent studies on the Winters sex ratio system in *Drosophila simulans*, which includes two drive loci and a suppressor locus, is consistent with this prediction [22]. When fecundity, fertility, or viability costs offset the effect of segregation distortion, purifying selection is expected to occur at the drive locus while background selection will occur at loci in linkage with it. DNA polymorphism is also reduced under background selection [33], but the resulting allele frequency distribution should be less skewed than under positive selection [34], [35]. If net selection is balancing, the allele frequency distribution is expected to be higher in the center and deficient in the tails when compared to neutral expectations [36].

Reed et al. [37] point out that in *Drosophila*, the selection coefficients attributable to drive appear to be strong, and speculate it is not a coincidence that drive is often found in areas of the X chromosome known to have both low recombination rates and low polymorphism. However, Derome et al. [38] found evidence of a very recent selective sweep around one recombining area of the X chromosome associated with the Paris drive system of *D. simulans*. Interestingly, recent evidence indicates that this drive system has subsequently declined in frequency in Madagascar due to the presence of a suppressor, indicating that the drive system is costly [39]. Furthermore, little sequence polymorphism was found on a drive X chromosome in *D. recens* by Dyer et al. [40], but their analysis suggested that the drive X chromosome in this species is no longer experiencing selective sweeps and is instead accumulating deleterious alleles and may be on its way to being lost. In a population genetic analysis of *segregation distorter (SD)*, an autosomal drive system in *D. melanogaster*, Presgraves et al. [41] found a widespread *SD* haplotype from Africa that contained no sequence variation across 39% of the second chromosome and attribute this result to a recent selective sweep in the last 3,000 years.

The stalk-eyed fly *Teleopsis dalmanni* provides an intriguing system for studying the effects of X chromosome drive on sequence variation. Multiple populations in Southeast Asia possess high frequencies of X chromosome drive, have close phylogenetic relationships [42], and exhibit variable degrees of reproductive isolation [43]. Limited recombination between *X^D^* and *X^ST^* chromosomes in *T. dalmanni* has been inferred to be caused by one or more inversions on the X chromosome associated with drive [44]. In the wild, drive persists in a natural polymorphism of *X^D^* and *X^ST^* chromosomes, sex ratios are strongly female-biased [45], and 8–25% of males produced strongly biased sex ratios [42]. The high frequency of drive in the wild suggests that meiotic drive in *T. dalmanni* is not nearing extinction and may be maintained by balancing selection [26]. Findings by Johns *et al.* [44] suggest that variation in drive is caused by Y-linked and autosomal suppressors and earlier studies report the presence of modifiers [5], [10]. In addition, crosses between allopatric and reproductively isolated populations have recently revealed the presence of cryptic drive (S. Christianson, C. Brand and G. Wilkinson, unpublished data). The combined presence of X chromosome drive, suppressors, modifiers and cryptic drive suggests a history of repeated selective sweeps. The recent discovery that *Teleopsis* flies carry a neo-X chromosome that is derived from the left arm of the second autosome in *Drosophila melanogaster* [46] provides additional motivation to examine patterns of sequence variation in these flies.

Here we use field and lab samples of *T. dalmanni* from sites in peninsular Malaysia and Sumatra to study the effects of X chromosome drive on patterns of DNA sequence variation. While the two populations readily interbreed in the lab, hybrid males are sterile while females are fertile [43]. We sequenced two regions approximately 50 cM apart on the X chromosome [44], [46] and two autosomal regions, which consist of one coding and one untranscribed region of the same gene. We then examine these sequences for patterns of polymorphism and consider the results in light of the predicted patterns left behind by different forms of selection.

## Materials and Methods

### Population samples

**Figure 1:**
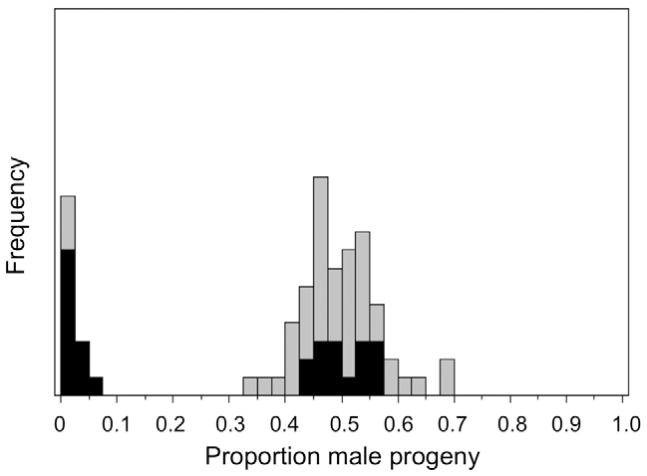
Brood sex ratios produced by 81 male *Teleopsis dalmanni* from the Gombak population. Highlighted individuals are used in this study and denoted *X^D^* if the progeny sex ratio is less than 0.1 male or *X^ST^* otherwise.

DNA sequences were obtained for a total of 50 *Teleopsis dalmanni* males. Thirteen were captured in August, 1999 near the Soraya research station in Aceh Province, Indonesia. Five of those males were returned to the lab alive, and after breeding to virgin females, produced offspring in a sex ratio not significantly different from 50∶50 (i.e. were non-drivers and were presumed to carry a standard X, *X^ST^*, chromosome). The remaining males died before they could be tested; all 13 were preserved in 70% ethanol. A second field-collected sample of 10 males was collected near the Gombak Field Research Center in September and October, 1989, in peninsular Malaysia. Each of these flies was captured in a separate aggregation at night, immediately frozen in liquid nitrogen (cf. Wilkinson and Reillo 1994), and subsequently stored at −20°C. In addition, we used 27 males with known progeny sex ratios from a laboratory population that contains descendants from 227 flies that were collected in August, 1999, from the Gombak site. At that time, 14 of 93 (15.0%) sons of field caught females exhibited significantly female-biased sex ratios [42]. The lab population has subsequently been maintained with approximately 2–4 overlapping generations per year and at least 100 breeding females. In June, 2002, males from the cage were bred to virgin females to identify carriers of the *X^D^* chromosome [29]. Out of 81 males tested by breeding to virgin females (mean ± SE number progeny/male = 94±7), 15 (18.5%) produced extremely female-biased sex ratios (mean ± SE male proportion = 0.013±0.005) and the rest produced unbiased sex ratios. We used 12 *X^D^* and 15 *X^ST^* of those males for this study (Fig. 1).

### Ethics statement

All necessary permits were obtained for the work described in this paper and include research permits from the Indonesian Institute of Science and from the Socio-Economic Research Unit of Malaysia. Additional permission to work at the Soraya field site was obtained from the Leuser Management unit in Medan and from the University of Malaysia in Kuala Lumpur to work at the Gombak Field Research Center. Flies were cultured in the lab under a permit from the USDA Animal and Plant Health Inspection Service. These studies do not involve endangered or protected species.

### DNA regions

Four DNA segments, two autosomal and two X-linked, were sequenced for this study. The autosomal regions included both a coding and an untranscribed region (UTR) of *bangles and beads* (*bnb*), the identity of which was inferred from a BlastX match (4e-36) between a 2000 bp *T. dalmanni* EST and *Drosophila melanogaster* reference protein sequence [47]. We amplified and sequenced approximately 700 bp of the coding region and 400 bp region of the UTR, which was approximately 620 bp 3′ from the sequenced portion of the coding region. Together these regions produced a total of 923 bp of aligned sequence after removing repetitive regions of variable length. We also amplified and sequenced two regions of the X chromosome, located approximately 50 cM apart in crosses involving flies only carrying *X^ST^* chromosomes [44], [46]. One region included about 1000 bp of a gene, *cryptocephal (crc)*, which varied in length due to the presence of an intron between positions 201 and 267 in the alignment and a tandemly repeated glutamine [48]. The other 680 bp region contained a dinucleotide microsatellite, ms125 [49]. We designed primers (Table 1) to amplify and sequence from either an EST for *crc* [47] or a 40 Kb sequence of a fosmid clone carrying *ms125* (R. Baker, unpublished data). In the final alignment the two X-linked loci totaled 1314 bp. Comparative genomic hybridization of male and female DNA confirmed that *bnb* is autosomal while *crc* is X-linked in *T. dalmanni* [46].

**Table 1:** Primer sequences and product sizes.

Locus	Primer sequence (5′ first)	Aligned product size
125	F: TGGTGTTAATGAACGAGTGACTTCF2: GAAGACTTGCATGAATGGCAF3: TGGTGTGCGTTTGCATTTATF4: TTCATTGCATTTGCATTCGR2: AAATGGAAAATTGTGGAAGTGGR3: GCACAAAACATGGCGAAAATR4: TGAAGAAAAATTGTATGAAATGAAAAGR5: GCCGCAGACATGACAGTAAA	460
*crc*	F2: ATCAAACCTTCGTCTCAGCtrrF: CCAGTTCAAATTGTAACCAACGR1: GCATAGAATTCACGTATAAGCGtrrR: TCGACAATTTGCATTTCACGTGC	712
*bnb*	F1: GAAACACCCGTAGAAGTTGTGCCAGR2: ACACGATGCGTATGTTGTGTGGGCR3: GGGGAAAACCTTAAGCCATTA	530
*bnb* UTR	F1: CAGAAGACCGGCAAGTAAATGSF2: TGCAAACAATGCTCAAGGACSF3: GGACGTTTCGAGGAAAGTGAR1: GATTTTTGCGACGGTTCAAG	391

DNA sequence chromatograms were manually edited using Chromas Lite (v2.01; Technelysium Pty Ltd.) and Sequencher v4, and corrected sequences aligned using Clustal W as implemented in Seaview v4 [50]. Haplotypes for X-linked loci were counted directly because males are haploid [51]. For autosomal loci, heterozygous individuals were identified by the presence of two nucleotides at one base position from both forward and reverse sequence reads. We excluded all base positions associated with repetitive DNA, indels and missing data from sequence analysis, but we used some of the information from repetitive regions in separate analyses (see below). All sequences used in this study are deposited in GenBank (XXXXX).

### Population genetic analysis

We counted the number of polymorphic sites (S) and singleton polymorphisms (S_i_) in the sequences, and used DnaSP v5.10.01 [52] to calculate several measures of polymorphism and divergence. For X-linked sequence we report the number of haplotypes (h) and two estimators of polymorphism: the average number of nucleotide differences within populations (π), and Watterson's [53] estimator of the proportion of segregating sites within populations (θ) for all sequences. For each region we also calculated two D statistics: Tajima's D [54] to detect departure from neutrality and Fu and Li's D* [55] to detect and differentiate between balancing and purifying selection. Any individuals that were missing sequence information were excluded from diversity calculations for X-linked or autosomal regions.

We also used the direct mode in DnaSP to perform four different HKA tests [56] to detect evidence of selection by comparing polymorphism and divergence between X-linked and autosomal sequence. In a conventional application of this test we compared the two field caught samples to determine if these regions show evidence of positive selection that might correspond to recent speciation. In addition, we make three additional comparisons to determine if patterns of polymorphism and divergence match random expectations when the captive males that either carried the X^D^ chromosome are compared to those that carried the X^ST^ chromosome, and when males from the field sample are compared to lab males containing either the X^D^ or the X^ST^ chromosome.

The relatively high mutation rates of repetitive DNA regions potentially provide additional information on polymorphism. The *ms125* sequence contained four different repetitive regions – the expected dinucleotide microsatellite, a polythymine repeat, a six-bp repeat, and an eight-bp repeat. In addition, the *crc* sequence contained a polyglutamine (CAA or CAG) repeat [48]. To assess variation at these repeat regions we counted the number of chromosomes containing identical sequences for each repeat region and then calculated haplotype diversity using the following formula:
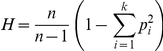
In this equation, *H* is haplotype diversity, *n* is the number of chromosomes sampled, *p_i_* is the frequency of the *i*th allele (which in this case is characterized by the number of repeats present at one of the five regions), and *k* is the number of alleles. In addition to calculating *H* for each repeat region, we also calculated *H* for each unique repeat combination formed by combining all repeats on each X chromosome.

## Results

**Figure 2:**
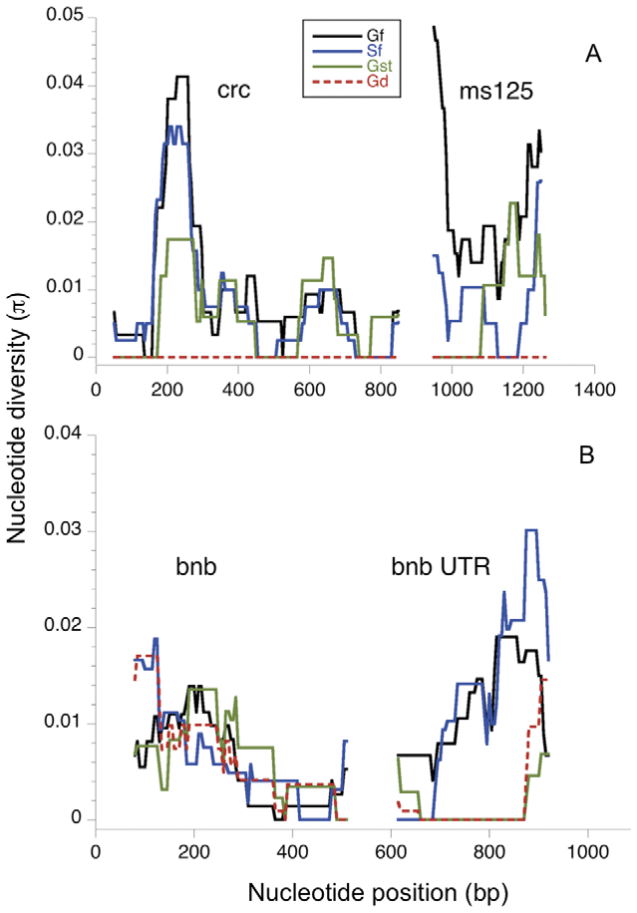
Nucleotide diversity for (A) the two X-linked regions and (B) the two autosomal regions. Sections containing variable repeats have been removed and estimates are from a sliding window with length 100 and step size 5 bp. An intron in *crc* occurs between 200 and 260 bp in the alignment. *Cryptocephal* and ms125 are separated by 50 cM on *X^ST^* chromosomes. The bnbUTR is approximately 620 bp 3′ from the sequenced portion of the coding region. Field collected samples are labeled Gf for Gombak and Sf for Soraya. Lab population samples are labeled Gst for Gombak males carrying *X^ST^* and Gd for Gombak males carrying *X^D^* as explained in Fig. 1.

**Figure 3:**
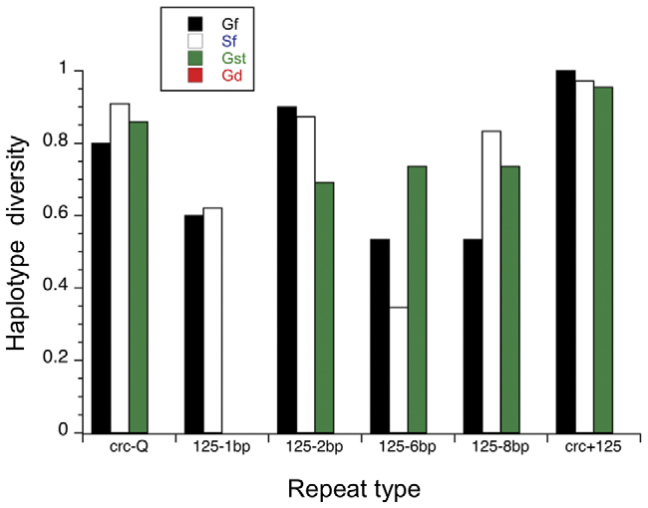
Haplotype diversity for each repetitive DNA region on the X chromosome by population. The final set of bars combines the glutamine repeat region in the *crc* locus with the four ms125 repeat regions to create a single haplotype for each individual. Sample labels as in Fig. 2. Note that the males containing an *X^D^* chromosome exhibit no haplotype variation.

Examination of aligned sequence data revealed a total of 48 autosomal and 73 X-linked segregating sites. The two field sampled populations showed very similar patterns of polymorphism across the X-linked (Fig. 2A) and autosomal (Fig. 2B) regions, with an increase in variation in the intronic region of *crc*. The lab population of males carrying an *X^ST^* chromosome exhibited patterns of autosomal and X-linked polymorphism that were similar to but lower than the field caught samples for parts of both X-linked and autosomal regions (Fig. 2). Examination of the estimates of sequence diversity and the number of singleton polymorphisms (Table 2) confirms that lab flies carrying the *X^ST^* chromosome have less than half the genetic variation in both X-linked and autosomal regions as field caught flies. However, the lab population males carrying the *X^D^* chromosome exhibited no polymorphism at either of the X-linked regions but had levels of variation at the autosomal locus, *bnb*, that were similar to those found in males carrying the *X^ST^* chromosome. Haplotype diversity among repeat regions of the X-linked markers differed among the four samples (Fig. 3) in a similar manner with Gombak *X^D^* males lacking any variation while males carrying an *X^ST^* chromosome from the lab contained levels of haplotype diversity comparable to the field caught samples from either population. After combining the data for all five repeats to create a single estimate of haplotype diversity, *H* equaled or was close to 1 for both field collected samples and the lab population of Gombak *X^ST^* males, but 0 for Gombak *X^D^* males.

**Table 2:** Summary of polymorphism and tests of neutrality by chromosomal region.

	X				Autosomal			
	Sor	Gom^89^	Gom^ST^	Gom^D^	Sor	Gom^89^	Gom^ST^	Gom^D^
n	8	6	6	9	22	14	24	22
S	41	45	14	0	28	29	13	13
S_i_	35	28	5	0	6	12	2	3
h	8	6	5	1	-	-	-	-
π†	0.95	1.51	0.60	0	0.89	0.79	0.42	0.40
θ†	1.22	1.55	0.47	0	0.83	1.09	0.38	0.39
D	−1.21	−0.14	0.66	‡	0.24	−1.27	0.37	0.09
D*	−1.53	−0.32	0.30	‡	0.22	−0.45	0.67	−0.10

† : multiplied by 10^−2^;

‡ : not calculated because of lack of polymorphism;

*P<0.05; n: number of chromosomes; S: number of nucleotide polymorphisms; S_i_: number of singleton polymorphisms; h: number of haplotypes (not available for autosomal sequences); π: average number of nucleotide differences between sequences; θ: Watterson's [53] estimator of the number of segregating sites between populations; D: Tajima's D; D*: Fu and Li's D.

The two measures of sequence diversity, π and θ, did not differ from each other for the autosomal or X-linked regions as indicated by the absence of any significant D or D* statistics (Table 2). These results provide evidence indicating that the *bnb* locus is not under strong selection and is therefore an appropriate locus to use for comparison to the X chromosome.

The HKA tests (Table 3) provide evidence consistent with recent positive selection on the *X^D^* chromosome. Comparison of polymorphism to divergence between the autosomal and X-linked regions for the field-collected samples from Gombak and Soraya revealed no significant difference (χ^2^ = 0.017, *P* = 0.896). Similarly, comparison of polymorphism to divergence between the X and autosomal regions using Gombak males collected in the field and nondrive males from the lab revealed no significant difference (χ^2^ = 0.046, *P* = 0.83) indicating that patterns of variation in the lab are comparable to those in the field. In contrast, an HKA test between X and autosomal regions using lab Gombak *X^D^* and *X^ST^* samples was significant (χ^2^ = 6.40, *P* = 0.012), as was an HKA test using the Gombak *X^D^* lab sample and the Gombak field sample (χ^2^ = 5.14, *P* = 0.023). Table 3 indicates that both significant test results arise from reduced polymorphism within the X-linked sequences of driving males relative to autosomal sequences and higher than expected sequence divergence for X-linked regions relative to autosomal regions. These results are consistent with a more recent coalescent time for loci on the *X^D^* chromosome than for the *X^ST^* chromosome.

**Table 3:** Levels of polymorphism and divergence (expected in parentheses) between autosomal and X-linked regions for different samples of males.

Sample comparison	Autosome	X
*Field: Gombak vs. Soraya*		
No. segregating sites (Gombak)	29 (29.9)	45 (44.1)
No. differences (Gombak vs. Soraya)	12.7 (11.8)	25.0 (25.9)
*Lab: drive vs. non-drive* *		
No. segregating sites (drive)	13 (8.8)	0 (4.2)
No. differences (drive *vs.* non-drive)	4.2 (8.4)	10.8 (6.6)
*Field vs. lab: Gombak field vs drive* *		
No. segregating sites (drive)	13 (8.6)	0 (4.4)
No. differences (field vs. drive)	14.5 (18.9)	20.8 (16.5)
*Field vs. lab: Gombak field vs nondrive*		
No. segregating sites (nondrive)	13 (13.7)	14 (13.3)
No. differences (field vs. nondrive)	15.0 (13.11)	27.7 (28.4)

*p<0.05; HKA test.

Comparison of the lab and field-collected X-linked sequences from the Gombak population reveals that one field-collected male had a sequence that was nearly identical to the *X^D^* haplotype identified in the lab population. Despite being collected 10 years previously, this individual exhibited only a single base pair difference from the lab *X^D^* haplotype. The divergence, i.e. the per site average number of nucleotide substitutions, between this individual and the *X^D^* haplotype is 0.076%. Repeat lengths were also identical with the exception of the dinucleotide repeat from the ms125 sequence, which differed by a single copy (Gombak drive males from the lab had 12 repeats while the field collected male had 11 repeats). In contrast, the haplotype of this individual had 17 fixed differences from all other field-collected Gombak sequences and a divergence of 2.18±0.37%. In comparison, the average divergence among the other field collected Gombak individuals was 1.28±0.22%. Based on this pattern of extreme similarity to drive and divergence from other field caught individuals, we infer that this individual also carried an *X^D^* chromosome.

## Discussion

In this study we identify a striking pattern of sequence polymorphism associated with X chromosome drive in *Teleopsis dalmanni* stalk-eyed flies. Males from a lab population that carry an *X^D^* chromosome contain typical levels of sequence variation at an autosomal region but lack sequence or repeat polymorphism at two X-linked regions that are estimated to be 50 cM apart on *X^ST^* chromosomes. In contrast, males from the lab population that do not carry an *X^D^* chromosome or males collected in the field from either of two populations contain appreciable variation among nucleotides and repeats on the X chromosome and do not exhibit significant differences in the pattern of variation across coding and noncoding regions of an autosomal locus. The presence of a single individual with a nearly identical haplotype in the Gombak field sample, which was collected 10 years prior to the formation of the lab colony, to the lab *X^D^* chromosome sequence provides compelling evidence that the drive haplotype persists in the field and exhibits considerable divergence (over 2%) from other field-collected sequences at these X-linked regions. This divergence suggests that the *X^D^* chromosome has been separated from the *X^ST^* chromosome for a considerable length of time, similar to what has been observed for *D. recens* [40].

One potential explanation for a reduction in genetic variation is a population bottleneck. However, a bottleneck should affect the entire genome, not just one class of X chromosome. Thus, given the presence of variation at autosomal loci in the same individuals a bottleneck in the wild or in the lab by itself is an insufficient explanation for the lack of polymorphism on the *X^D^* chromosome. Captive rearing has, though, had a demonstrable effect on the amount of genetic variation present in the lab population. The amount of sequence diversity at the X-linked and autosomal regions in the Gombak field-collected sample is more than double what is present in the nondrive lab sample (Table 3). The reduction in variation is, however, comparable in magnitude for the X-linked and autosomal regions.

Another potential demographic factor that could influence genetic diversity is the effective population size of the X chromosome relative to the autosomes. A population with an unbiased sex ratio will have four copies of each autosome for every three X chromosomes and the autosomes should, therefore, support more genetic diversity, on average. Data from this study are not consistent with this prediction given that there is as much, if not more, variation among the X-linked as the autosomal regions for the field collected samples. However, because wild populations of *T. dalmanni* are female-biased [45], reduced effective population size of the X chromosome should be less of a factor in this species than in species with unbiased population sex ratios [49].

Instead of demographic processes, a combination of selection and reduced recombination provides a better explanation for the lack of polymorphism on the Gombak *X^D^* chromosome. The HKA tests revealed evidence for positive selection on the Gombak *X^D^* chromosome when compared to the Gombak *X^ST^* chromosomes from the lab or field. When contrasted with the result of the HKA test comparing field collected Gombak and Soraya samples, it is apparent that selection has been concentrated on the chromosome that carries drive. Sequence analysis of additional chromosomal regions from drive and nondrive individuals are needed to determine where and how selection is acting. The presence of a nearly identical X haplotype from an individual collected 10 years earlier is consistent with a past selective sweep on the *T. dalmanni X^D^* chromosome that occurred sufficiently recently, given the population size of these flies, to prevent variation in sequence or repeats from accumulating. In this regard, these results are similar to those reported by Derome et al. [38] who found no variation at two drive-associated regions in *D. simulans* from Madagascar. While it is hypothetically possible that the drive haplotype underwent a selective sweep in captivity, we find such a scenario unlikely given that the frequency of drive among X chromosomes sampled from field caught females was 15% when the lab population was started [42] and four years later the frequency of the drive haplotype in the population cage was nearly the same, i.e. 18.5% [29].

The effect of a selective sweep on reducing variation is dependent on the rate of recombination. In regions of low recombination, genetic hitchhiking should reduce DNA polymorphism more effectively [30], [57]. The results from this study are consistent with studies from *Drosophila*, which indicate that regions of reduced DNA polymorphism are more often caused by selective sweeps [58]–[61] than by demographic effects [62] or background selection [63]. In particular, several species in the genus show low sequence polymorphism in chromosomal regions with reduced recombination [63]–[69].

**Figure 4:**
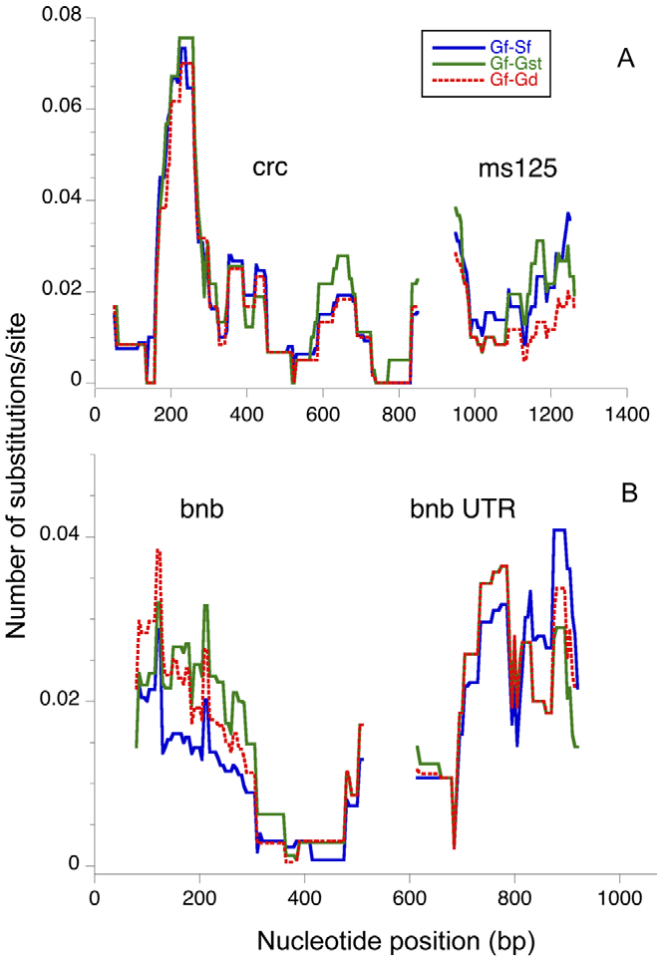
Nucleotide divergence between the field-collected Gombak population and each of the other three samples. Labels as in Fig. 2 for (A) the two X-linked regions and (B) the two autosomal regions.

While a selective sweep is expected to eliminate linked neutral variation, it is not predicted to affect genetic divergence [30], [32]. Indeed, comparison of patterns of average sequence divergence between field and lab flies is independent of the presence of drive (Fig. 4). However, examination of the observed and expected values in the HKA tests (Table 3) show that there is more divergence than expected at X-linked sequences when comparing drive to nondrive males and less divergence than expected at autosomal sequences using both field and lab-reared flies. The usual assumption underlying models of genetic hitchhiking is that an organism gains a fitness benefit from a favorable mutation acting in isolation. But meiotic drive systems can involve drive, target, modifier and suppressor loci, and evolution at one locus in effect changes the environment of the other loci. This is the essence of intragenomic conflict, which can create antagonistic coevolution similar to what occurs between the sexes within a species [70], [71]. Unlike simple positive selection, antagonistic coevolution between loci is expected to promote rapid divergence between populations and possibly drive speciation [72].

Meiotic drive has, therefore, the potential to cause divergence and reproductive isolation between species. Because meiotic drive is associated with sperm production, it has been hypothesized to be associated with male hybrid sterility [73], [74]. Drive-suppressor systems have been shown to evolve rapidly within populations [38], [39], [75], [76] and diverge from the standard X chromosomes of the same species [40], [77]. The presence of considerable, i.e. 2%, sequence divergence between the *X^D^* and *X^ST^* chromosomes indicate that the drive chromosome must have been genetically isolated from the *X^ST^* chromosome for many years. Nevertheless, the lack of sequence or tandem repeat polymorphisms on the *X^D^* chromosome is consistent with a recent selective sweep, as expected if an arms race among drive chromosome variants [29] is ongoing. While these results do not demonstrate that sex chromosome meiotic drive has caused reproductive isolation between populations of *T. dalmanni* [43], they are consistent with studies on *D. simulans* and *D. pseudoobscura*, where genomic conflicts involving meiotic drive have been linked to reproductive isolation [16], [78]–[81]. Furthermore, the recent discovery of cryptic drive in stalk-eyed flies derived from backcrosses between reproductively isolated populations (S. Christianson, C. Brand and G. Wilkinson, unpublished data) suggests that genome scans of sequence diversity have the potential to reveal signatures of drive and suppression, even among flies that do not currently exhibit drive.
